# A cross-omics integrative study of metabolic signatures of chronic obstructive pulmonary disease

**DOI:** 10.1186/s12890-020-01222-7

**Published:** 2020-07-16

**Authors:** Ivana Prokić, Lies Lahousse, Maaike de Vries, Jun Liu, Marita Kalaoja, Judith M. Vonk, Diana A. van der Plaat, Cleo C. van Diemen, Ashley van der Spek, Alexandra Zhernakova, Jingyuan Fu, Mohsen Ghanbari, Mika Ala-Korpela, Johannes Kettunen, Aki S. Havulinna, Markus Perola, Veikko Salomaa, Lars Lind, Johan Ärnlöv, Bruno H. C. Stricker, Guy G. Brusselle, H. Marike Boezen, Cornelia M. van Duijn, Najaf Amin

**Affiliations:** 1 Department of Epidemiology, Erasmus Medical Center, Rotterdam, The Netherlands; 2 Department of Bioanalysis, Pharmaceutical Care Unit, Ghent University, Ghent, Belgium; 3 Department of Epidemiology, University of Groningen, University Medical Center Groningen, Groningen, The Netherlands; 4 Groningen Research Institute for Asthma and COPD (GRIAC), University of Groningen, University Medical Center Groningen, Groningen, The Netherlands; 5 Nuffield Department of Population Health, University of Oxford, Oxford, UK; 6 Computational Medicine department, Center for Life Course Health Research, Biocenter Oulu, University of Oulu, Oulu, Finland; 7 National Heart and Lung Institute, Imperial College London, London, UK; 8 Department of Genetics, University of Groningen, University Medical Center Groningen, Groningen, The Netherlands; 9 Department of Pediatrics, University of Groningen, University Medical Center Groningen, Groningen, The Netherlands; 10 Department of Genetics, School of Medicine,, Mashhad University of Medical Sciences, Mashhad, Iran; 11 NMR Metabolomics Laboratory, School of Pharmacy, University of Eastern Finland, Kuopio, Finland; 12 Finnish Institute for Health and Welfare, Helsinki, Finland; 13 Molecular Metabolism Research Program, Faculty of Medicine, University of Helsinki, Helsinki, Finland; 14 Department of Medical Sciences, Uppsala University, Uppsala, Sweden; 15 Division of Family Medicine and Primary Care, Department of Neurobiology, Care Sciences and Society, Karolinska Institute, Huddinge, Sweden; 16 School of Health and Social Sciences, Dalarna University, Falun, Sweden; 17 Department of Respiratory Medicine, Ghent University Hospital, Ghent, Belgium; 18 Department of Respiratory Medicine, Erasmus Medical Center, Rotterdam, The Netherlands

**Keywords:** COPD, Metabolomics, Mendelian randomization, Glycoprotein acetyls, Biomarkers

## Abstract

**Background:**

Chronic obstructive pulmonary disease (COPD) is a common lung disorder characterized by persistent and progressive airflow limitation as well as systemic changes. Metabolic changes in blood may help detect COPD in an earlier stage and predict prognosis.

**Methods:**

We conducted a comprehensive study of circulating metabolites, measured by proton Nuclear Magnetic Resonance Spectroscopy, in relation with COPD and lung function. The discovery sample consisted of 5557 individuals from two large population-based studies in the Netherlands, the Rotterdam Study and the Erasmus Rucphen Family study. Significant findings were replicated in 12,205 individuals from the Lifelines-DEEP study, FINRISK and the Prospective Investigation of the Vasculature in Uppsala Seniors (PIVUS) studies. For replicated metabolites further investigation of causality was performed, utilizing genetics in the Mendelian randomization approach.

**Results:**

There were 602 cases of COPD and 4955 controls used in the discovery meta-analysis. Our logistic regression results showed that higher levels of plasma Glycoprotein acetyls (GlycA) are significantly associated with COPD (OR = 1.16, *P* = 5.6 × 10^− 4^ in the discovery and OR = 1.30, *P* = 1.8 × 10^− 6^ in the replication sample). A bi-directional two-sample Mendelian randomization analysis suggested that circulating blood GlycA is not causally related to COPD, but that COPD causally increases GlycA levels. Using the prospective data of the same sample of Rotterdam Study in Cox-regression, we show that the circulating GlycA level is a predictive biomarker of COPD incidence (HR = 1.99, 95%CI 1.52–2.60, comparing those in the highest and lowest quartile of GlycA) but is not significantly associated with mortality in COPD patients (HR = 1.07, 95%CI 0.94–1.20).

**Conclusions:**

Our study shows that circulating blood GlycA is a biomarker of early COPD pathology.

## Background

Chronic obstructive pulmonary disease (COPD) is a progressive inflammatory lung disease and currently the third leading cause of death worldwide [1, 2]. COPD is characterised by chronic airway inflammation, airway remodelling and airflow limitation [3]. A reduced ratio of the Forced Expiratory Volume in 1 s (FEV_1_) to Forced Vital Capacity (FVC) is a measure of obstruction and is used to diagnose COPD even before the onset of clinical symptoms [3, 4]. Smoking is the most important risk factor for COPD and related to impaired lung function [2]. COPD is a complex heterogeneous disease in which systemic features beyond airflow obstruction, including systemic inflammation, oxidative stress, muscle dysfunction, cachexia and vascular pathology occur [5, 6]. Understanding these systemic effects may give new insights into the pathogenesis and progression of COPD but may alternatively yield important clues for preventive research.

Recent developments in metabolomics have made it possible to investigate the associations between circulating metabolites and COPD. Glycoprotein acetyls (GlycA) was found to be predictive for several chronic diseases, among which COPD [7]. In a previous metabolomics study using proton Nuclear Magnetic Resonance (^1^H-NMR), lower levels of lipoproteins, N,N-dimethylglycine and higher levels of glutamine, phenylalanine, 3-methylhistidine and ketone bodies were found in the circulation of ex-smoking COPD patients compared with ex-smoking controls [8]. In severe COPD patients, branched chain amino acids (BCAAs) were found to be lower, compared with controls [8]. Interestingly, BCAAs, 3-methylhistidine, ketone bodies, and triglycerides were negatively correlated with cachexia and positively correlated with systemic inflammation [8], but these findings have not been replicated. Another question that remains to be answered is whether the metabolic changes are a cause or a consequence of COPD. If the latter is true, the metabolites may be relevant for the disease progression and prognosis.

To answer these questions, we performed a comprehensive integrative metabolic analysis to identify plasma metabolic measures associated with COPD and lung function levels, defined as FEV_1_/FVC, using the NMR approach in a set of large epidemiological studies, in depth characterized for genetic and environmental risk factors. The discovery phase of the study was conducted in two population-based studies in the Netherlands, the Rotterdam Study (RS) [9] and the Erasmus Rucphen Family study (ERF) [10, 11]. A replication meta-analysis was conducted in the Lifelines-DEEP study (LLDEEP) [12], two cohorts of the FINRISK study [13, 14] and the Prospective Investigation of the Vasculature in Uppsala Seniors (PIVUS) study [15, 16].

## Methods

### Study population

#### Studies included in the discovery sample

The RS is a population-based study of 14,926 people older than 45 years, from the Ommoord area of Rotterdam, incorporating three independent cohorts: RS-I (established in 1989), RS-II (2000) and RS-III (2006), with multiple subsequent visits [9]. Participants filled in questionnaires, underwent physical examination and provided fasting blood samples at each visit. For this analysis, three independent samples from different RS cohorts were enrolled: Sample 1) visit 4 of RS-I (RS-I-4); sample 2) a combined sample, which we collectively call RS-E5 in this manuscript, comprising of visit 5 of RS-I (RS-I-5), visit 3 of RS-II (RS-II-3), and visit 2 of RS-III (RS-III-2); and sample 3) another independent set from RS-III-2.

ERF is a population-based study from the south-west of the Netherlands. It is a genetically isolated population comprising 3465 living descendants of 22 couples from the nineteenth century and their spouses [10]. The baseline data collection was performed in 2002–2005 when participants underwent physical examinations, provided blood samples and completed questionnaires. A follow-up of the participants was performed in 2015–2018, reviewing the medical records at the general practitioner’s office.

#### Studies included in the replication sample

LLDEEP is a sub-cohort of the large general population-based cohort study Lifelines, which was initiated to study genes, exposures and their interactions in the aetiology of complex multifactorial diseases and healthy ageing [17, 18]. LLDEEP consists of 1500 participants who registered at the Lifelines research site in Groningen between April and August 2013. These subjects gave additional biological materials, including blood samples for metabolite and inflammation profiling, and extensive phenotype information [12]. Metabolic and lung function data were available for 717 LLDEEP individuals and these subjects are included in the current study.

The FINRISK cohorts comprise cross-sectional population surveys that are carried out every 5 years since 1972, to assess the risk factors of chronic diseases (e.g. cardio-vascular disease, diabetes, obesity, cancer) and health behaviour in the working age population (25–74 years of age), in 3–5 large study areas of Finland. The FINRISK surveys are conducted by the National Institute for Health and Welfare, THL (previously National Public Health Institute, KTL). Extensive information from each participant was collected at baseline via questionnaire and health examination with blood collection. The cohorts were followed up by linking them to national health registers. The cohorts FINRISK 1997 (total of 6898 participants) and an extension of FINRISK 2007, known as DIetary, Lifestyle and Genetic determinants of Obesity and Metabolic syndrome (DILGOM) study [19] (total of 4600 participants) are included in our replication sample for COPD analysis.

The PIVUS study started in 2001 with the aim to investigate endothelial function as a prospective cardiovascular risk factor in elderly subjects. A random sample of Uppsala city residents were invited from the register of inhabitants within 1 month following their 70th birthday. No exclusion criteria were applied except that participants were required to have a Swedish identification number. In PIVUS, 1016 subjects agreed to participate, resulting in a participation rate of 50.1% of all invited, whereof 51.5% were female. The participants have undergone a range of physical measurements, and given information about their medical history, lifestyle habits and regular medication. In addition, blood samples were drawn.

### Assessment of COPD status and lung function measurements

COPD in the RS was defined as pre-bronchodilator FEV_1_/FVC < 0.7, assessed either by spirometry at the RS research center or by reviewing medical histories of the participants. Spirometry was performed in the RS by trained paramedical personnel, according to the guidelines of the American Thoracic Society/European Respiratory Society (ATS/ERS). When spirometry measurements were absent or uninterpretable, all files from specialists and general practitioners were reviewed to set a diagnosis of COPD. In total, this analysis included 541 incident COPD subjects and 4407 subjects without COPD which had metabolomics data available from all three RS cohorts.

For the ERF study, the doctor’s diagnosis of COPD was confirmed by reviewing medical records based on FEV_1_/FVC < 0.7, with or without medication use. If the information on FVC was missing, the following criteria for COPD were used: FEV_1_ < 80% of predicted, use of respiratory medication and a COPD diagnosis mentioned in the report of the respiratory specialist to the general practitioner. In total, 61 incident and prevalent COPD subjects and 548 subjects without COPD which had metabolomics data available were included from ERF study. For ERF participants, we did not have lung function measurements at the time of the metabolomics measurements, so we did not include this cohort in the FEV_1_/FVC analysis.

For LLDEEP, COPD was also defined as a FEV_1_/FVC < 0.7. Pre-bronchodilator spirometry was performed according to the ATS/ERS guidelines using a Welch Allyn Version 1.6.0.489, PC-based Spiroperfect with CA Workstation. Technical quality and results were assessed by well-trained assistants and abnormal results were re-evaluated by lung physicians.

In the FINRISK study the COPD information was extracted based on diagnoses and reimbursement information from the National health register, which include the Drug Reimbursement Register, the Care Register for Health Care, the Register for Prescribed Drug Purchases, the Causes-of-Death Register, and the Cancer Register. The maximum retrospective time period available for obtaining prevalent disease events was 20 years for DILGOM and 10 years for FINRISK97.

In the PIVUS study FEV_1_ and FVC were assessed with spirometry using a Vitalograph Alpha spirometer (Vitalograph Ltd. Buckingham, United Kingdom) according to the American Thoracic Society recommendations [20, 21]. The best value of three acceptable recordings was used. FEV_1_ and FVC expressed as percent of predicted values, were adjusted for age, sex and height according to Hedenström’s formula [22, 23]. PIVUS study was included only in the FEV_1_/FVC analysis, as this study does not have confirmed diagnosis of COPD by lung specialist.

### Assessment of blood metabolites

Metabolic profiling in RS, ERF and LLDEEP was done as part of the 4th Rainbow Project of the BioBanking for Medical Research Infrastructure of the Netherlands (BBMRI-NL) (https://www.bbmri.nl/omics-metabolomics/). For all studies used in the discovery and replication samples, to quantify the metabolite biomarkers random selection of fasting EDTA plasma samples were used for quantitative high-throughput ^1^H-NMR metabolomics platform performed by the same company using the same standardized quality control protocol (Nightingale Ltd., Helsinki, Finland). All samples were stored at − 80 °C which ensures the biological stability. Details of the protocol and advantages of the NMR-based metabolomics analyses using plasma were described elsewhere [24, 25]. The protocol describes steps for quality control and sample preparation, data storage and spectral analyses. If metabolite values were flagged to be unreliable by the quality control protocol, they were treated as missing. If distributions of the metabolites deviated from normal, every cohort applied normalization steps as suggested by Nightingale. Those included natural logarithm transformation and scaling to standard deviation units. Using this method, we were able to quantify a wide range of blood metabolite biomarkers such as lipoprotein fractions, amino-acids, cholesterol levels, glycerides, phospholipids, fatty acids, ketone bodies and metabolites related to inflammation and glycolysis. In total, 161 metabolites, overlapping between RS and ERF, were used in the discovery analysis.

### Statistical analyses

#### Association of COPD and FEV_1_/FVC with metabolites

Per cohort, we used transformed metabolite levels as independent variable and COPD status or FEV_1_/FVC as dependent variables in logistic and linear regression models, respectively. The models were adjusted for age, sex, body mass index (BMI, kg/m^2^), lipid lowering medication (LLM) use and smoking status (current, ex- or never smokers). For the discovery sample, the results from ERF, RS-I-4, RS-E5 and RS-III-2 were meta-analysed using fixed effect models in “*METAL*” software [26]. As the metabolites are known to be highly correlated, we applied the method by Li and Ji [27] to assess the number of independent metabolites. Using this method, we calculated that for the 161 metabolites, the number of independent tests was 45, which resulted in the Bonferroni significance threshold of *P* = 0.001 (0.05/45). Significant metabolites were further tested for replication in the meta-analysis of LLDEEP, FINRISK1997 and DILGOM studies for the COPD analysis and of LLDEEP and PIVUS studies for the FEV_1_/FVC analysis. Again, the same regression models were used for the fixed effect meta-analysis in “*METAL*” software.

For significant metabolites, we calculated the odds ratios per quartile of the metabolite distribution in the discovery sample. To investigate the effects of smoking on this association, we used two logistic regression models, one adjusted for age, sex, BMI and LLM use, and a second model additionally adjusted for smoking status (current, ex- and never smokers). Results from each cohort were combined using inverse-variance weighted fixed effects meta-analysis in “*rmeta*” package in R.

#### Exploring causality of the association between COPD and metabolites

We have used a bi-directional approach in which we examined whether: 1) the genetic determinants of the significant metabolites are associated with COPD and lung function, which would lead to the conclusion that the metabolites are most likely driving the disease; 2) the genetic determinants of COPD are associated with significant metabolites when the metabolites would most likely be altered as an integral part of the disease pathophysiology and may be biomarkers. The R package “*TwoSampleMR*” was used for the two sample Mendelian Randomization (MR) tests [28, 29]. We used the genetic information from previously published genome-wide association studies (GWAS) on metabolites (Model 1) [25] and COPD (Model 2) [30]. In brief, the genetic score was based on the top single nucleotide polymorphisms (SNPs, *P*-value < 5 × 10^− 8^) with linkage disequilibrium (LD) R^2^ < 0.05 within 500 kb clumping distance. Harmonization was checked, including the strand issues and palindromic SNPs. It resulted in eight independent SNPs for COPD (R^2^ = 1.7%), and nine SNPs for GlycA (R^2^ = 2.3%). Inverse variance weighted MR, Maximum likelihood MR, MR Egger analysis and median-based estimator were performed to check the significant results.

#### Association with morbidity and mortality

We wanted to investigate whether an identified metabolite in the circulation is a biomarker of early pathology thus can be used as a predictive or diagnostic biomarker or rather prognostic biomarker for mortality in COPD patients. To this end, we performed an analysis in the Rotterdam Study in which we associated identified metabolite to the future risk of COPD. We determined the relative risk by quartile of the metabolite concentration in the circulation, using the lowest quartile as a reference. Only incident patients are included in this analysis (whole RS sample, in total 541 case and 4407 controls); prevalent COPD patients are excluded. To investigate whether metabolites have utility in predicting COPD, we constructed classical receiver operating curves (ROC) and compared areas under the curve (AUC) [31]. To further investigate whether the identified metabolites may act as biomarker of the disease prognosis, we performed a survival analysis in SPSS, similar to the previous study by Fischer and colleagues for all-cause mortality, ignoring any underlying morbidity [32]. To check whether the metabolites associated with mortality in COPD patients, we performed the Cox proportional hazards model in three RS cohorts. Analyses were adjusted for age at sampling, sex and smoking. We further performed a similar analysis using four quartiles of metabolite, testing in COPD cases and controls.

## Results

### Descriptive characteristics of the samples

Descriptive characteristics of all cohorts used in the analysis are presented in Table 1. Comparing the discovery cohorts, ERF participants were younger (mean age 49.0 ± 13.3) and had a higher percentage of current smokers compared to the participants of the three RS cohorts (RS-I-4 mean age 74.8 ± 6.5; RS-E5 mean age 68.4 ± 5.7; RS-III-2 mean age 62.8 ± 5.8). The RS cohorts had a higher percentage of users of the LLM, compared to ERF (Table 1).

**Table 1:** Discovery population characteristics per cohort

Study	Discovery cohorts				Replication cohorts			
	ERF	RS-I-4	RS-E5	RS-III-2	LLDEEP	FINRISK97	DILGOM	PIVUS
**N**	609	2777	686	1485	717	6898	4600	854
**Age, mean (sd)**	49.0 (13.3)	74.8 (6.5)	68.4 (5.7)	62.8 (5.8)	46.0 (14.3)	48.0 (13.1)	52.3 (13.5)	70 (0)
**Women, % (n)**	55.8 (340)	58.2 (1615)	57.6 (395)	57.8 (859)	56.3 (404)	51.6 (3561)	53.4 (2458)	48.2 (412)
**COPD cases, % (n)**	10.0 (61)	12.1 (336)	10.3 (71)	9.0 (134)	13.8 (99)	0.6 (43)	0.8 (35)	NA
**FEV**_**1**_**/FVC, mean (sd), % of all**	NA	0.73 (0.08), 48.8	0.76 (0.07), 91.3	0.77 (0.07), 91.9	0.77 (0.08), 100	NA	NA	0.76 (0.11), 100
**BMI, mean (sd)**	27.2 (4.85)	27.4 (4.1)	27.8 (4.3)	27.4 (4.5)	25.4 (4.1)	26.6 (4.5)	27.2 (4.8)	27.1 (4.26)
**Current smokers, % (n)**	43.3 (264)	12.6 (349)	9.5 (65)	13.7 (203)	20.5 (147)	23.9 (1648)	17.6 (810)	10.2 (87)
**Ex-smokers, % (n)**	30.0 (183)	56.1 (1559)	57.0 (391)	50.2 (746)	NA	22.9 (1577)	26.3 (1210)	41.5 (354)
**Never smokers, % (n)**	26.6 (162)	31.3 (869)	33.5 (230)	36.1 (536)	79.4 (570)	53.2 (3673)	56.1 (2580)	48.2 (412)
**Pack-years of smoking, mean (sd), % of all**^**a**^	24.9(20.4) 72.7	24.2 (23.4), 64.7	22.0 (20.8) 66.3	19.5 (20.3) 63.8	NA	NA	NA	NA
**LLM users, % (n)**	12.3 (75)	22.4 (621)	32.5 (223)	22.2 (329)	3.9 (28)	3.4 (237)	15.7 (721)	16.5 (141)

*sd* standard deviation, *RS-E5* consists of RS-I-5, RS-II-3 and RS-III-2; ^a^ Pack-years calculated in current and ex-smokers only, so “% of all” excludes never smokers; *LLM* lipid-lowering medication, *NA* not applicable

The mean FEV_1_/FVC and BMI were comparable across the studies. Descriptive characteristics for COPD cases and subjects without COPD separately in the discovery cohorts are provided in eTable 1 in the Supplement. In general, COPD subjects were older and more often smokers compared to subjects without COPD. Since FINRISK97 and DILGOM studies are based on the data from National health registers, and thus do not have minimum age entry criteria, the percentage of COPD cases is lower compared with discovery sample, containing elderly population.

### Association of COPD and FEV_1_/FVC with metabolites

**Fig. 1:**
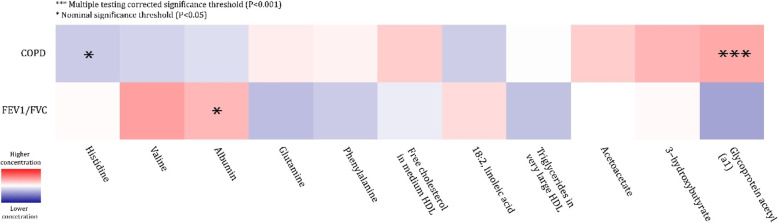
Top metabolites associated with COPD and/or FEV_1_/FVC. Colors represent standardized effect estimates of the metabolite association with corresponding trait (COPD, FEV_1_/FVC). Red color means that the trait is associated with a higher metabolite concentration, while blue represents a lower metabolite concetration. For replicated metabolites, replication *P*-value is shown with stars: *P < 0.05 and ***P < 0.001. HDL – high-density lipoprotein

In the discovery sample, six plasma metabolites were associated with COPD at a significance level of 5% (Table 2**,** Fig. 1).

**Table 2:** Metabolites associated with COPD in the discovery and replication studies

Metabolite	Discovery meta-analysis						Replication meta-analysis					
	β	SE	OR	***P***-value	Direction^**a**^	N	β	SE	OR	***P***-value	Direction^**b**^	N
GlycA	0.152	0.044	1.16	**5.6 × 10**^**−4**^	++++	5557	0.266	0.053	1.30	**1.8 × 10**^**−6**^	+++	12,205
3-hydroxybutyrate	0.122	0.041	1.13	0.003	++++	5002	−0.031	0.057	0.97	0.662	− − +	12,173
Histidine	−0.097	0.047	0.91	0.037	− − − −	5534	−0.153	0.063	0.86	0.020	−−−	12,200
Free cholesterol in med. HDL	0.099	0.049	1.10	0.045	+ − ++	5557	0.004	0.063	1.00	0.867	− − +	12,208
Acetoacetate	0.084	0.042	1.09	0.047	++ − +	5551	−0.061	0.059	0.94	0.360	−−−	12,204
18:2, linoleic acid	−0.095	0.048	0.91	0.049	+ −−−	5546	−0.036	0.057	0.96	0.238	+ − +	12,167

Model adjusted for age, sex, BMI, LLM use and smoking status; *GlycA* Glycoprotein acetyls, *HDL* high density lipoprotein, *β* effect size, *SE* standard error, *OR* odds ratio; Direction - direction of the effect in individual studies; N - meta-analysis sample size; ^a^ Direction of the effect in the discovery studies in order: ERF, RS-III-2, RS-E5, RS-I-4; ^b^ Direction of the effect in the replication studies in order: LLDEEP, FINRISK97, DILGOM; In bold: significant results (*P* < 0.001)

At nominal significance, higher levels of GlycA (odds ratio (OR) = 1.16; *P* = 5.6 × 10^− 4^), 3-hydroxybutyrate (OR = 1.13; *P* = 0.003), free cholesterol in medium high-density lipoprotein (HDL, OR = 1.10; *P* = 0.045) and acetoacetate (OR = 1.09; *P* = 0.047) were associated with a higher prevalence of COPD. Higher levels of histidine and 18:2 linoleic acid (OR = 0.91 for both, *P* = 0.04 and *P* = 0.05 respectively) were associated with a lower prevalence of COPD. When considering the multiple testing correction threshold, only GlycA was significantly associated with COPD (*P* = 5.6 × 10^− 4^). We tested all six metabolites for replication in the independent samples. The association of higher levels of GlycA with COPD was significantly replicated (OR = 1.30, *P* = 1.8 × 10^− 6^) in the 12,205 participants of the replication sample, after multiple testing correction.

Findings for the FEV_1_/FVC ratio were not consistent over the discovery and replication studies. Adjusting for multiple testing, we found in the discovery cohorts that lower levels of valine (*β* = 0.005, *P* = 2.5 × 10^− 4^) and higher levels of GlycA (*β* = − 0.005, *P* = 4.5 × 10^− 4^) were associated with a lower FEV_1_/FVC ratio (Table 3, Fig. 1).

**Table 3:** Top metabolites associated with FEV_1_/FVC - Results of the discovery and replication studies

Metabolite	Discovery meta-analysis					Replication meta-analysis				
	β	SE	***P***-value	Direction^**a**^	N	β	SE	***P***-value	Direction^**b**^	N
Valine	0.005	0.001	**2.5 × 10**^**–4**^	+++	3324	− 0.0015	0.0023	0.5314	−+	1460
GlycA	−0.005	0.001	**4.5 × 10**^**–4**^	–––	3324	− 0.0010	0.0022	0.6438	––	1463
Albumin	0.004	0.001	0.0047	+++	3324	0.0045	0.0021	0.0353	++	1463
Glutamine	−0.003	0.001	0.0097	–––	3323	0.0029	0.0023	0.1923	++	1393
Triglycerides in very large HDL	−0.003	0.001	0.0160	–––	3324	0.0031	0.0022	0.1491	++	1469
Phenylalanine	−0.003	0.001	0.0334	–––	3324	− 0.0012	0.0023	0.5899	+−	1450

Model adjusted for age, sex, BMI, LLM use and smoking status; *HDL* high density lipoprotein, *β* effect size, *SE* standard error; Direction - direction of the effect in individual studies; N - meta-analysis sample size; ^a^ Direction of the effect in the discovery studies in order: RS-III-2, RS-E5, RS-I-4; ^b^ Direction of the effect in the replication studies in order: LLDEEP, PIVUS; In bold: significant results (*P* < 0.001)

Other metabolites that reached nominal significance in the discovery included albumin which was positively associated with FEV_1_/FVC, and glutamine, triglycerides in very large HDL and phenylalanine which were negatively associated with FEV_1_/FVC (Table 3**,** Fig. 1). Only the association of FEV_1_/FVC to albumin showed nominal significance in the replication samples (*β* = 0.005, *P =* 0.03), but none were significantly associated when considering multiple testing correction. Meta-analysis results of all metabolites tested for the association with COPD and FEV_1_/FVC in the discovery sample are provided in the supplementary material (eTable 4 and eTable 5, respectively).

### Exploring causality of COPD and circulating GlycA

Next, we performed a Mendelian Randomisation experiment investigating the hypothesis that: 1) GlycA is increasing the risk of COPD and therefore the genetic determinants of GlycA (used as instrumental variables) are also associated with COPD and 2) the opposite scenario is true in which (pre)clinical COPD pathology increases GlycA levels. The results of both models are presented in Table 4**.**

**Table 4:** Results of the bi-directional MR approach on GlycA and COPD

Model	Exposure	Outcome	R^**2**^	nSNP	Method	β	SE	***P***-value
1	GlycA	COPD	2.30	9	Inverse variance weighted	0.001	0.027	0.97
					Weighted median	−0.009	0.029	0.76
					Weighted mode	−0.028	0.039	0.49
					Simple mode	−0.013	0.046	0.79
					MR Egger	−0.140	0.147	0.37
2	COPD	GlycA	1.72	8	Inverse variance weighted	0.306	0.090	**0.00068**
					Weighted median	0.348	0.115	**0.0024**
					Weighted mode	0.378	0.150	**0.04**
					Simple mode	0.359	0.157	0.06
					MR Egger	0.412	0.624	0.53

R^2^ - the explained variance in the exposure by applied genetic risk score; nSNP - number of SNPs used to construct the genetic risk score; β - the weighted effect of the genetic risk score of exposure on outcome; *SE* standard error; Significance threshold = *P*-value < 0.05. The Egger regression is a test to check the assumption of the instrument strength being independent of the direct effect to the outcome and should be *P* > 0.05

The genetic risk score (GRS) for Model 1 included nine independent SNPs (R^2^ = 2.3%) and yielded no significant evidence for association (*P* = 0.97 for inverse variance weighted method). In Model 2, we found that genes associated with a higher risk of COPD are also associated with higher levels of GlycA, through the COPD (Table 4, *P* = 0.00068 for inverse variance weighted method), suggesting that COPD pathology increased GlycA levels. The results of weighted median and weighted mode were significant as well (*P*-value< 0.05). This analysis is based on eight independent SNPs in the GRS (R^2^ = 1.7%). No heterogeneity or pleiotropic effect were detected. Leaving out either SNP did not change the significance of the MR results. The detailed MR output are shown in supplementary information.

### Is circulating GlycA predictive biomarker for COPD?

Compared to the lowest quartile, those subjects in the highest quartile of GlycA had a 1.99-fold (95% Confidence interval: 1.52–2.60) higher risk of developing COPD, after adjustment for age, sex, BMI and LLM use (eTable 2). Smoking accounted for a part of the observed association between plasma GlycA and COPD attenuating the OR for those in the highest quartile of GlycA to 1.74, while the association remained significant (95% Confidence interval: 1.32–2.28). To test whether circulating GlycA adds to the predictive value, we compared the AUC curves for the models including: 1) age and sex (AUC = 0.601); 2) age, sex and smoking (AUC = 0.670) and 3) age, sex, smoking and circulating GlycA levels in blood (AUC = 0.675). The AUC comparing model 2 and 1 shows that smoking is associated with an increase in AUC by 0.069. Adding circulating GlycA increased the AUC further by only 0.005 (eFigure 1).

### Is circulating GlycA a prognostic biomarker for mortality in COPD?

A previous study has shown that GlycA is a predictor of all-cause mortality in the general population [32]. We confirm this in our current study, after adjustment for age, sex and smoking (hazard ratio (HR) = 1.16, *P* = 4.39 × 10^− 9^) (eTable 3). The mean follow-up time in years was 6.94, ranging from 0.04 to 15.96. We first performed the analysis with continuous GlycA and then compared mortality across the quartiles of GlycA. We found that those in the highest quartile have 1.4-fold (95% Confidence interval: 1.22–1.61, *P* = 1.64 × 10^− 6^) higher risk of mortality during follow-up compared to those in the lowest quartile (eTable 3). However, when stratifying these analyses by COPD status, we observed that this association is driven by controls (eTable 3; eFigure 2). In COPD patients, circulating GlycA levels are not significantly associated with mortality when studying GlycA as a continuous variable (HR = 1.06, *P* = 0.32) nor for those in the highest quartile (HR = 1.07, *P* = 0.70 in COPD cases). In those without COPD, the association of continuous GlycA to mortality is stronger and significant (HR = 1.18, *P* = 1.43 × 10^− 9^).

## Discussion

In our metabolome-wide discovery analysis, we identified 11 plasma metabolites associated with COPD or lung function levels (FEV_1_/FVC) at marginal significance. Of these 11 metabolites, only higher levels of GlycA were significantly associated with COPD when adjusting for multiple testing and this is the only metabolite we could replicate in the independent cohorts. Our MR analysis suggested a causal relation between COPD and higher GlycA levels in the circulation by showing that the genetic predisposition to COPD associates with GlycA. The GlycA level seemed to be an early biomarker of COPD since it was associated with the incidence of COPD, even after adjustment for smoking. Although GlycA was found to be a predictor of mortality in the general population [33], the metabolite did not predict mortality in COPD patients.

GlycA is the most convincing and interesting finding of our study. This metabolite was recently associated with the incidence of a variety of disorders, including COPD based on record linkage [7]. Using two population-based cohorts, we identified new associations with GlycA including alcoholic liver disease, chronic renal failure, glomerular diseases and inflammatory polyarthropathies. The GlycA associations were for a large part independent of that of high-sensitivity C-reactive protein (hsCRP), but GlycA and hsCRP also share contributions to mortality risk, suggesting chronic inflammation as the common pathway. GlycA is shown to be a biomarker for chronic inflammation, neutrophil activity and risk of future severe infection, even superior compared with CRP [34, 35].

The present study extends previous research by widening the number of NMR metabolites studied and we found that GlycA is the only metabolite significantly associated with COPD after adjusting for multiple testing. Our analyses were adjusted for smoking and the association between GlycA and COPD is thus not explained by smoking. We used data integration approach (MR) to test the hypothesis that GlycA increases the risk of COPD causally or rather is a bystander biomarker that is part of the disease pathogenesis (marker of the disease). Our findings suggest that the latter is more likely, as the genes associated with COPD also associate with GlycA levels. In contrast, no support was found for the hypothesis that GlycA is a causal determinant of COPD: the genes that are known to be associated with GlycA levels are not associated with the risk of COPD. The findings of the MR are in line with the finding that GlycA was not consistently associated with the FEV_1_/FVC ratio across the discovery and replication cohorts, which suggests that GlycA is more likely increased as an early consequence of the developed disease. This is in line with other studies on different diseases involving systemic inflammation. However, as many other factors, aside from genetics, play a role in this complex disease and blood metabolic patterns, our MR results need further corroboration using experimental animal models to support the causality.

Although it is known to be a marker of acute inflammation, it has also been shown that it is predictive of long-term risk of severe infection, and high levels correlated with an increased risk of hospitalization and death from septicaemia and pneumonia [34]. This is particularly important for exacerbations of COPD and the prognosis. In the present paper we do not find evidence that GlycA is associated with COPD mortality. Such relationship was seen for cardiovascular disease. GlycA not only increased the risk of incident cardiovascular disease [7, 36] but was also associated with a 5-fold increased 12-year risk of mortality in those with the highest GlycA levels [7]. This suggests that our analysis would benefit from increasing the sample size even more.

GlycA, is a composite NMR-based signal related to changes in multiple circulating glycoproteins, mainly orosomucoids [37], which are a positive acute phase proteins, and their concentration increases in response to systemic tissue injury, inflammation or infection [38, 39]. Even in apparently healthy people high GlycA was related with elevation in many inflammatory cytokines suggesting they may be in a state of chronic inflammatory response up to 10 years [34]. Another acute phase protein modulating the immune response, whose deficiency has an established effect on COPD pathogenesis, is alpha 1-antitrypsin (AAT). It is found that although alpha-1-acid-glycoprotein had a strongest correlation with GlycA, it was the AAT variation that had the most predictive properties for morbidity and mortality for many different diseases [39]. Moreover, protein haptoglobin, also included in GlycA signal, was estimated to be the strongest predictor of chronic lower respiratory diseases of all proteins included in this signal [39]. GlycA is mainly produced by the liver, but it is also synthesized in myelocytes and released by activated neutrophils [40]. Being a type I acute phase protein, GlycA is induced by cytokines, interleukins and tumour necrosis factor alpha (TNFα) [41, 42], which among others stimulate a systemic inflammatory response in COPD patients who lose weight [43]. GlycA is one of the main drug binding proteins, carrying basic and neutral lipophilic drugs such as steroid hormones or medications in blood [44].

A strength of our study is that it is the largest and most comprehensive metabolic study of COPD and lung function. Another strength is the use of the NMR platform, which is valued for being non-invasive, non-destructive, fast and for providing highly reproducible results [45]. A limitation of this study is our COPD definition, mainly based on pre-bronchodilator lung function measurements or review of medical records and national registries, which may have introduced some selection bias. Nevertheless, we do identify and replicate significant results which should be further corroborated in studies with post-bronchodilator measures. Our MR approach allowed us to gain more insight into the direction of the effects, suggesting that GlycA is an independent biomarker of COPD. Yet we have to acknowledge that MR is limited to the knowledge of the genetic determinants of both COPD and GlycA. In addition, we acknowledge possible limitations of MR due to pleiotropy, the lack of trans-ethnic studies and remaining bias due to canalization.

## Conclusions

Altogether, combining the epidemiological data with our MR analyses suggests that GlycA is a biomarker of COPD inflammatory pathways, present in higher concentrations even before the COPD is clinically present. Further studies should investigate the possibility for GlycA to serve as a prediction tool for COPD morbidity and severity. Further functional studies investigating the role of GlycA in COPD will provide more insight into the pathogenesis, prognosis and treatment response of patients with COPD. Our study highlights the power of cross-omics and epidemiological data integration.

## Supplementary information

**Additional file 1.**

## Data Availability

The datasets generated and analysed during the current study are not publicly available due to the stringent consent form requirements signed by the study participants, but are available from the corresponding author on reasonable request.

## Abbreviations

hsCRP: High-sensitivity CRP
1H-NMR: Proton Nuclear Magnetic Resonance
ATS: American thoracic society
ATT: Alpha 1-antitrypsin
AUC: Area under the curve
BCAAs: Branched chain amino acids
BMI: Body mass index
COPD: Chronic Obstructive Pulmonary Disease
CRP: C-reactive protein
DILGOM: DIetary, Lifestyle and Genetic determinants of Obesity and Metabolic syndrome study
ERF: Erasmus Rucphen Family study
ERS: European respiratory society
FEV_1_: Forced Expiratory Volume in 1 s
FVC: Forced Vital Capacity
GlycA: Glycoprotein-acetyls
GRS: Genetic risk score
GWAS: Genome-wide association study
HR: Hazard ratio
LLDEEP: Lifelines Deep study
LLM: Lipid lowering medication
MR: Mendelian Randomization
OR: Odds ratio
PIVUS: Prospective Investigation of the Vasculature in Uppsala Seniors study
ROC: Receiver operating curves
RS: Rotterdam Study
SNP: Single nucleotide polymorphism
TNFα: Tumor necrosis factor alpha
