# Supplementary material for: A cross-omics integrative study of metabolic signatures of chronic obstructive pulmonary disease
Source: BMC Pulm Med. 2020 Jul 16;20:193. doi: 10.1186/s12890-020-01222-7 (PMC7364599; doi:10.1186/s12890-020-01222-7)
Supplement: Supplementary file 1 — Additional file 1. [file 12890_2020_1222_MOESM1_ESM.zip › Supplementary material I ProkicR1.docx]

**Supplementary material of manuscript:**

**A cross-omics integrative study of metabolic signatures of Chronic Obstructive Pulmonary Disease**

Ivana Nedeljkovic^1^, Lies Lahousse^1,2^, Maaike de Vries^3,4^, Jun Liu^1,5^, Marita Kalaoja^6^, Judith M. Vonk^3,4^, Diana A. van der Plaat^3,4,7^, Cleo C. van Diemen^8^, Ashley van der Spek^1^, Alexandra Zhernakova^8^, Jingyuan Fu^8,9^, Mohsen Ghanbari^1,10^, Mika Ala-Korpela^6,11^, Johannes Kettunen^6,12^, Aki S. Havulinna^12,13^, Markus Perola^12,13^, Veikko Salomaa^12^, Lars Lind^14^, Johan Ärnlöv^15,16^, Bruno H.C. Stricker^1^, Guy G. Brusselle^1,17,18^, H. Marike Boezen^3,4^, Cornelia M. van Duijn^1,5^*, Najaf Amin^1,5^*

Full results of the MR analysis are available here:

| **eTable 1. Discovery population characteristics per cohort and by COPD status categories** | | | | | | | | | | | | |
| --- | --- | --- | --- | --- | --- | --- | --- | --- | --- | --- | --- | --- |
| **Study** | **ERF** | | | **RS-I-4** | | | **RS-E5** | | | **RS-III-2** | | |
| **COPD status** | **COPD cases** | **Controls** | **All** | **COPD cases** | **Controls** | **All** | **COPD cases** | **Controls** | **All** | **COPD cases** | **Controls** | **All** |
| **N** | 61 | 548 | 609 | 336 | 2441 | 2777 | 71 | 615 | 686 | 134 | 1351 | 1485 |
| **Age, mean (sd)** | 58.7 (10.0) | 48.0 (13.2) | 49.0 (13.3) | 76.2 (6.3) | 74.6 (6.5) | 74.8 (6.5) | 69.5 (4.9) | 68.3 (5.7) | 68.4 (5.7) | 63.4 (6.3) | 62.7 (5.8) | 62.8 (5.8) |
| **Women, % (n)** | 60.7 (37) | 55.3 (303) | 55.8 (340) | 42.6 (143) | 60.3 (1472) | 58.2 (1615) |  | 58.9 (362) | 57.6 (395) | 44.8 (60) | 59.1 (799) | 57.8 (859) |
| **FEV1/FVC, mean (sd), % of all** | - | - | - | 0.61 (0.07), 63.7 | 0.75 (0.06), 46.7 | 0.73 (0.08), 48.8 | 0.63 (0.07), 91.5 | 0.78 (0.06), 91.2 | 0.76 (0.07), 91.3 | 0.63 (0.07), 97.8 | 0.78 (0.05), 91.3 | 0.77 (0.07), 91.9 |
| **BMI, mean (sd)** | 27.0 (5.8) | 27.2 (4.7) | 27.2 (4.85) | 26.8 (4.2) | 27.5 (4.1) | 27.4 (4.1) | 27.2 (3.9) | 27.9 (4.3) | 27.8 (4.3) | 26.6 (4.2) | 27.5 (4.5) | 27.4 (4.5) |
| **Current smokers, % (n)** | 62.3 (38) | 41.2 (226) | 43.3 (264) | 21.7 (73) | 11.3 (276) | 12.6 (349) | 18.3 (13) | 8.5 (52) | 9.5 (65) | 35.1 (47) | 11.5 (156) | 13.7 (203) |
| **Ex-smokers, % (n)** | 37.7 (23) | 29.2 (160) | 30.0 (183) | 63.4 (213) | 55.1 (1346) | 56.1 (1559) | 57.7 (41) | 56.9 (350) | 57.0 (391) | 46.3 (62) | 50.6 (684) | 50.2 (746) |
| **Never smokers, % (n)** | 0 | 29.6 (162) | 26.6 (162) | 14.9 (50) | 33.6 (819) | 31.3 (869) | 23.9 (17) | 34.6 (213) | 33.5 (230) | 18.7 (25) | 37.8 (511) | 36.1 (536) |
| **Pack-years of smoking, mean (sd), % of all^a^** | 37.35 (26.5) 100.0 | 22.9 (18.5) 69.7 | 24.9(20.4) 72.7 | 34.3 (27.2) 81.3 | 22.4 (22.2) 62.4 | 24.2 (23.4), 64.7 | 34.7 (19.0) 76.1 | 20.3 (20.5) 65.2 | 22.0 (20.8) 66.3 | 30.5 (27.6) 81.3 | 18.0 (18.7) 62.1 | 19.5 (20.3) 63.8 |
| **Lipid lowering medication users, % (n)** | 23.0 (14) | 11.1 (61) | 12.3 (75) | 22.3 (75) | 22.4 (546) | 22.4 (621) | 33.8 (24) | 32.4 (199) | 32.5 (223) | 23.9 (32) | 22.0 (297) | 22.2 (329) |
| RS-E5: consists of RS-I-5, RS-II-3 and RS-III-2; ^a^ Pack-years calculated in current and ex-smokers only, so "% of all" excludes never smokers | | | | | | | | | | | | |

| **eTable 2. The risk of COPD per four quartiles of GlycA concentration** | | | | | | | |
| --- | --- | --- | --- | --- | --- | --- | --- |
|  |  |  | **COPD** | | **Total** | **OR1 [95% CI]** | **OR2 [95% CI]** |
|  | **Quartiles** |  | **Control** | **Case*** |  |  |  |
| **Glycoprotein acetyls** | **1st** | **N** | 1154 | 109 | 1263 | Reference | Reference |
|  |  | **%** | 91.4 | 8.6 | 100 |  |  |
|  | **2nd** | **N** | 1108 | 132 | 1240 | 1.40 [1.07-1.84] | 1.35 [1.03-1.78] |
|  |  | **%** | 89.4 | 10.6 | 100 |  |  |
|  | **3rd** | **N** | 1089 | 137 | 1226 | 1.54 [1.17-2.02] | 1.40 [1.06-1.84] |
|  |  | **%** | 88.8 | 11.2 | 100 |  |  |
|  | **4th** | **N** | 1056 | 163 | 1219 | 1.99 [1.52-2.60] | 1.74 [1.32-2.28] |
|  |  | **%** | 86.6 | 13.4 | 100 |  |  |
|  | **Total** | **N** | 4407 | 541 | 4948 | 1.23 [1.12-1.34] | 1.18 [1.07-1.29] |
|  |  | **%** | 89.1 | 10.9 | 100 |  |  |
| *Only incident cases included from RS. OR: Odds ratio for the increase in COPD risk per one standard deviation increase of Glycoprotein acetyls (GlycA) concentration. OR1 is from the model 1, adjusted for age, sex, BMI and lipid lowering medication; OR2 is from the model 2, additionally adjusted for smoking status. | | | | | | | |
|  |  |  |  |  |  |  |  |
|  |  |  |  |  |  |  |  |
|  |  |  |  |  |  |  |  |

| **eTable 3. Cox proportional hazard analysis for GlycA in total sample and stratified by COPD status** | | | | | | | | | | | | | | | | |
| --- | --- | --- | --- | --- | --- | --- | --- | --- | --- | --- | --- | --- | --- | --- | --- | --- |
|  |  | **Whole sample** | | | | | **COPD cases** | | | | | **COPD controls** | | | | |
|  |  | **HR** | **95%CI** | **95%CI** | **P-value** | **N** | **HR** | **95%CI** | **95%CI** | **P-value** | **N** | **HR** | **95%CI** | **95%CI** | **P-value** | **N** |
|  |  |  | **lower** | **upper** |  |  |  | **lower** | **upper** |  |  |  | **lower** | **upper** |  |  |
| GlycA - total | | 1.159 | 1.103 | 1.218 | **4.39×10^-9^** | 4923 | 1.063 | 0.943 | 1.198 | 0.318 | 535 | 1.182 | 1.12 | 1.248 | **1.43×10^-9^** | 4388 |
| GlycA-quartiles | 1st |  | | | | 1245 |  | | | | 109 |  | | | | 1136 |
|  | 2nd | 1.063 | 0.923 | 1.225 | 0.397 | 1245 | 0.8 | 0.541 | 1.181 | 0.261 | 131 | 1.106 | 0.95 | 1.288 | 0.192 | 1114 |
|  | 3rd | 1.127 | 0.98 | 1.296 | 0.094 | 1204 | 0.91 | 0.639 | 1.297 | 0.602 | 134 | 1.159 | 0.995 | 1.349 | 0.058 | 1070 |
|  | 4th | 1.404 | 1.222 | 1.614 | **1.64×10^-6^** | 1229 | 1.07 | 0.757 | 1.512 | 0.703 | 161 | 1.476 | 1.268 | 1.718 | **5.05×10^-7^** | 1068 |
| HR: hazard ratio; 95%CI lower: lower endpoint of the 95% confidence interval for HR; 95%CI upper: upper endpoint of the 95% confidence interval for HR; N: sample size; GlycA: alpha-1 Glycoprotein acetyls. | | | | | | | | | | | | | | | | |
|  |  |  |  |  |  |  |  |  |  |  |  |  |  |  |  |  |

**eTable 4 and eTable 5 are provided as excel tables.**

**eFigure 1.**
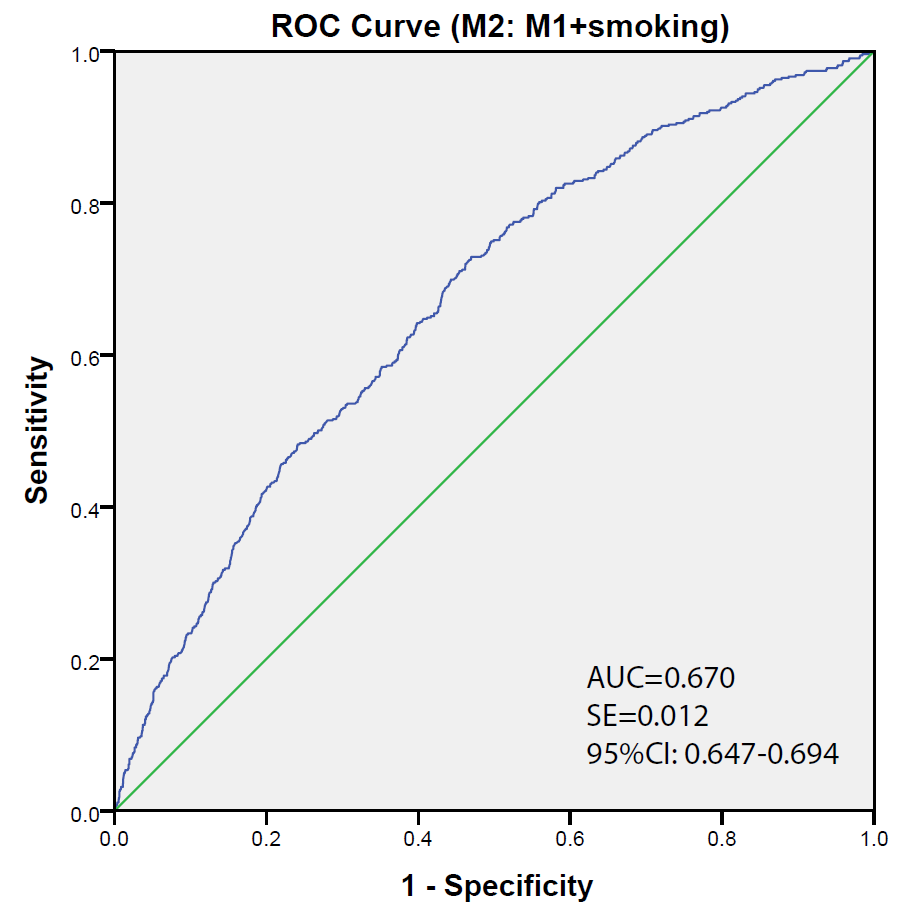

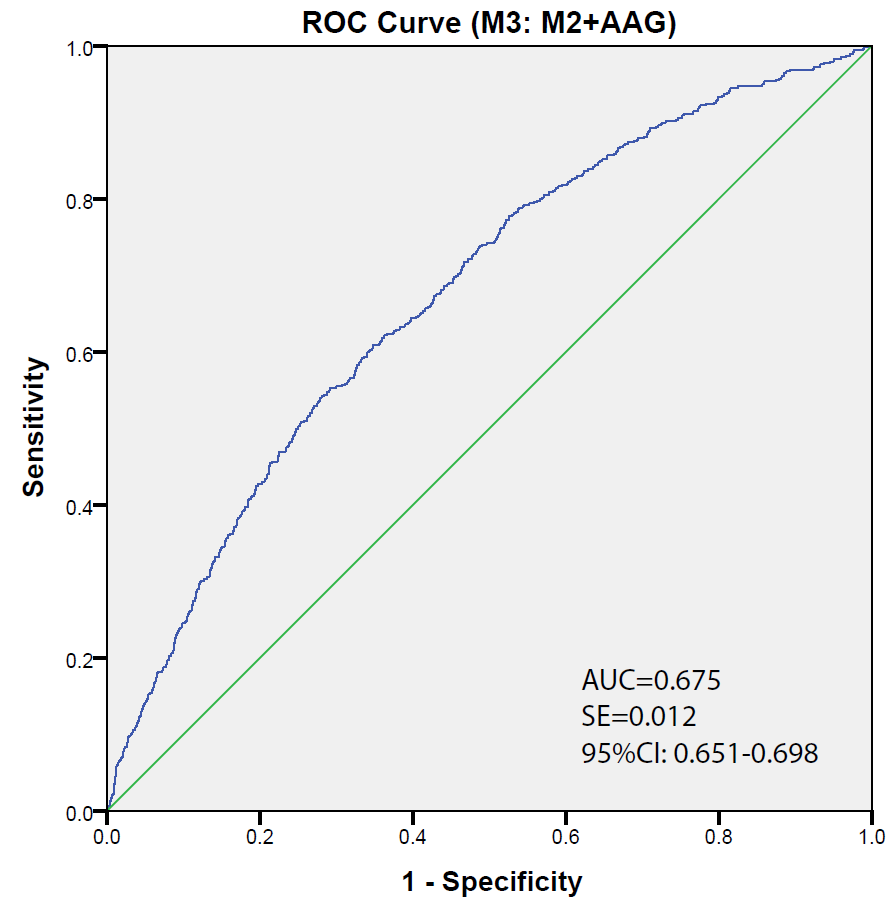

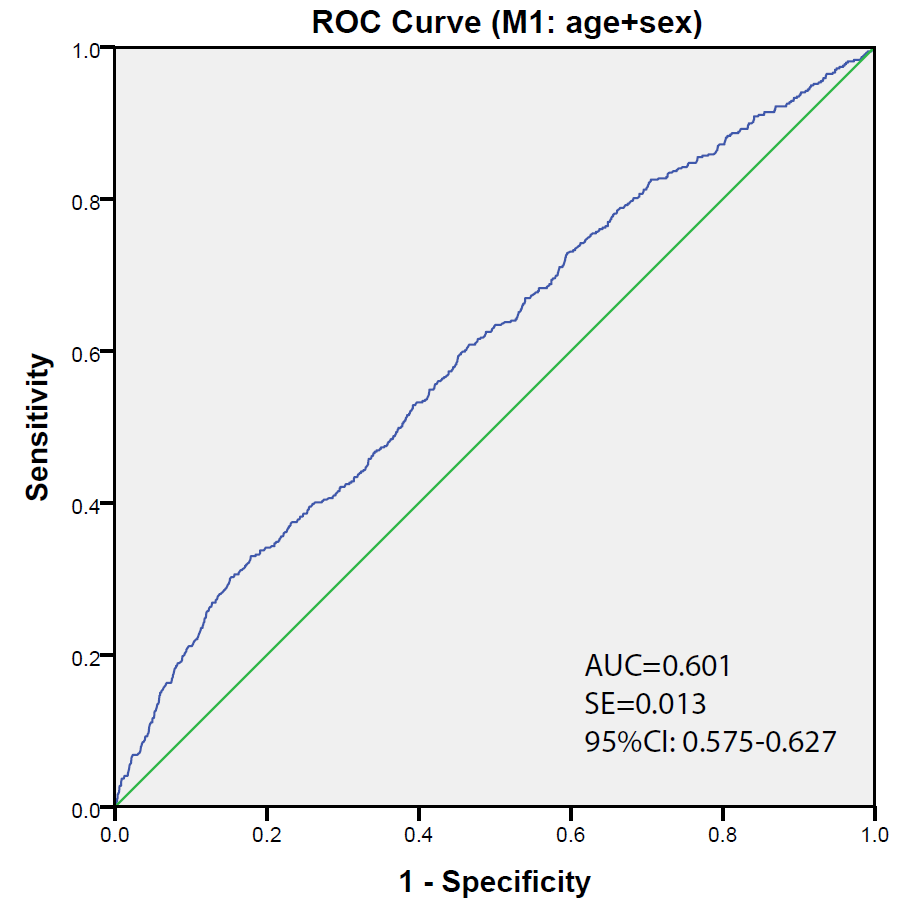


**eFigure 1. Comparison of Area Under the Curves (AUC) for three models.** ROC: receiver operating characteristic; Model 1: COPD~age+sex; Model 2: COPD~age+sex+smoking; Model 3: COPD~age+sex+smoking+GlycA; SE: standard error; CI: confidence interval.

**eFigure 2.**


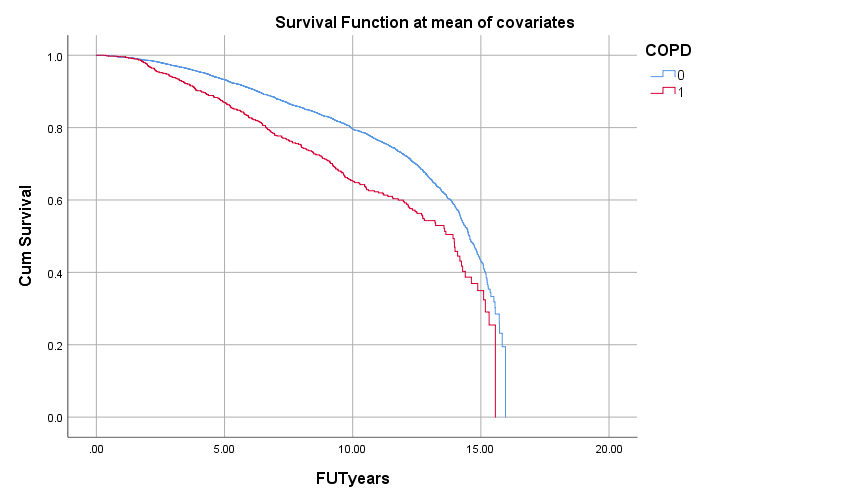


**eFigure 2. Kaplan-Meier survival curve.** CumSurvival: Cumulative survival; FUTyears: Follow-up time in years; The curves are showing cumulative survival of COPD patients (in red) versus controls (in blue) over the follow-up period (mean (FUT)=6.91years).
